# Supplementary material for: Identification and functional analysis of non-coding regulatory small RNA FenSr3 in Bacillus amyloliquefaciens LPB-18
Source: PeerJ. 2023 May 15;11:e15236. doi: 10.7717/peerj.15236 (PMC10194069; doi:10.7717/peerj.15236)
Supplement: Supplemental Information 4 [file peerj-11-15236-s004.zip › KO/CK-vs-T1_map/map05226.html]

KEGG PATHWAY: Gastric cancer - Reference pathway


|  |  |
| --- | --- |
| **Gastric cancer - Reference pathway** |  |

[
Pathway menu
| Organism menu
| Pathway entry
| Show description
| User data mapping
]

Gastric cancer (GC) is one of the world's most common cancers. According to Lauren's histological classification gastric cancer is divided into two distinct histological groups - the intestinal and diffuse types. Several genetic changes have been identified in intestinal-type GC. The intestinal metaplasia is characterized by mutations in p53 gene, reduced expression of retinoic acid receptor beta (RAR-beta) and hTERT expression. Gastric adenomas furthermore display mutations in the APC gene, reduced p27 expression and cyclin E amplification. In addition, amplification and overexpression of c-ErbB2, reduced TGF-beta receptor type I (TGFBRI) expression and complete loss of p27 expression are commonly observed in more advanced GC. The main molecular changes observed in diffuse-type GCs include loss of E-cadherin function by mutations in CDH1 and amplification of MET and FGFR2F.

|  |  |  |
| --- | --- | --- |
| Reference pathway | 184% 150% 122% 100% 82% 67% 55% | 图片下载 |
